# When the Past Catches Up: A Case of Latent Neurocysticercosis Presenting With Seizures

**DOI:** 10.7759/cureus.102638

**Published:** 2026-01-30

**Authors:** Kanwal Rashid, Amy Chen, Tzipora Levitt, Rashmika Mohunsing, Rachel Siegel, Abubakar Farooq, Adesh Ramdass

**Affiliations:** 1 Internal Medicine, Icahn School of Medicine at Mount Sinai, Queens Hospital Center, New York, USA; 2 Medicine, New York Institute of Technology, New York, USA; 3 Medicine, College of Osteopathic Medicine, New York Institute of Technology, New York, USA; 4 Medicine, St. George’s University, New York, USA; 5 Research and Development, ClinRe, Arlington, USA; 6 Medicine, Fatima Memorial Hospital, Lahore, PAK; 7 Medicine, Icahn School of Medicine at Mount Sinai, Queens Hospital Center, New York, USA

**Keywords:** antiparasitic therapy, calcified granulomas, central nervous system parasitosis, immigrant health, neurocysticercosis, new-onset seizures, ring-enhancing lesions, taenia solium

## Abstract

Neurocysticercosis (NCC), a central nervous system infection caused by the larval form of *Taenia solium*, remains one of the leading causes of acquired epilepsy worldwide, particularly among individuals from endemic regions. We describe a 41-year-old man originally from Guatemala who presented with new-onset generalized tonic-clonic seizures nearly 20 years after immigration to the United States, without recent travel or new exposure risks. Brain imaging demonstrated multiple calcified lesions and peripherally enhancing cystic structures consistent with NCC. This case is distinctive due to the prolonged latency between presumed exposure and symptom onset, as well as a seronegative presentation in which diagnosis relied primarily on characteristic neuroimaging findings. The report underscores the importance of detailed epidemiologic history-taking and maintaining diagnostic vigilance for parasitic infections when evaluating new-onset seizures after remote exposure.

## Introduction

Neurocysticercosis (NCC) is a neurologic disease caused by the larval stage of *Taenia solium*, also known as the “pork tapeworm” [1]. NCC is endemic in regions such as Latin America, sub-Saharan Africa, and parts of Asia, where it accounts for up to 30% of epilepsy cases [2]. While humans are the definitive host in intestinal taeniasis, NCC occurs when humans act as accidental intermediate hosts after ingesting *T. solium* eggs via fecal-oral transmission, leading to the development of cysticerci within the central nervous system (CNS) [2,3].

Although data on NCC incidence in the United States are limited, it is estimated that 1,500-2,500 new cases occur annually, with a substantial proportion requiring hospitalization [4]. Over a 10-year period (2003-2012), more than 18,000 hospitalizations for NCC were recorded in national inpatient data, illustrating its substantial burden on the U.S. healthcare system [5]. NCC disproportionately affects immigrants from endemic regions and is likely underdiagnosed due to limited clinician awareness and misattribution of seizures to more common causes [6].

Patients with NCC can remain asymptomatic for years after initial infection, as viable cysticerci typically provoke minimal immune response from the host [3]. A long latency period between exposure and symptom onset is common, with symptoms usually manifesting only when cysts reach the degenerative stage, causing perilesional inflammation and clinical manifestations such as seizures, headaches, or focal neurological deficits [7]. Over time, cysticerci may involute or calcify, leaving nonviable granulomas visible on neuroimaging [3,6]. These lesions may remain epileptogenic due to intermittent perilesional edema and inflammation, which can trigger seizures even in the absence of viable cysts [8,9].

While classic risk factors include pork consumption and residence in endemic areas, NCC should also be considered in patients without these exposures, particularly among immigrants [4]. We present a case of a healthy adult male from Guatemala who developed two new-onset seizures 18 years after immigration, leading to the diagnosis of NCC on neuroimaging.

## Case presentation

A 41-year-old male with no significant past medical history presented to the Emergency Department (ED) after experiencing two witnessed generalized tonic-clonic seizures on the day of presentation. The first seizure occurred during sleep and was described by his wife as generalized shaking with frothing at the mouth and jaw clenching, followed by postictal confusion and fatigue. A second seizure occurred four hours later, prompting ED presentation. The patient and his wife denied tongue biting, urinary incontinence, fever, chills, headache, visual changes, nausea, vomiting, or diarrhea. He reported no prior neurological symptoms and denied alcohol, tobacco, or illicit drug use, as well as any allergies. A CT head performed in the ED demonstrated lesions suspicious for NCC, and the patient was admitted for further evaluation.

The patient immigrated to the United States from Guatemala in 2007. Since immigration, he had not traveled outside of the U.S. and had not consumed pork. His only notable past medical and surgical history was lumbar spine surgery in 2011 for a herniated disc, with recent recurrence of neuropathic pain treated with prednisone.

On admission, the patient was a well-developed, well-nourished male in no acute distress. Vital signs were within normal limits. Neurological examination showed the patient to be alert and oriented ×3, with intact cranial nerves II-XII, normal strength and tone in all extremities, and symmetric deep tendon reflexes. Sensory examination was unremarkable, and coordination testing was normal. The only abnormal finding on physical examination was a positive right-sided straight leg raise test, consistent with his known lumbar radiculopathy.

Laboratory studies demonstrated normal leukocyte count and electrolytes. Liver function tests were unremarkable aside from mildly elevated ALT. HIV and hepatitis C virus screens were negative. Further workup revealed stool ova and parasite testing positive for moderate *Blastocystis hominis* cysts and a few *Endolimax nana* cysts. Tests for *Strongyloides* antibody, Quantiferon-TB, and cysticercosis antibody were negative. Ophthalmology examination prior to therapy was negative for intraocular cysts (Table 1).

**Table 1:** Laboratory values. RBC: red blood cell, WBC: white blood cell, Hb: hemoglobin, MCV: mean corpuscular volume, ALT: alanine aminotransferase, AST: aspartate aminotransferase, ALP: alkaline phosphatase, TB: tuberculosis, HIV: human immunodeficiency virus

Component	Results	Reference range
WBC	7.34 K/uL	4.80 - 10.80 x 10 (3)/mcL
RBC	5.64 M/uL	4.70 - 6.10 x 10 (6)/mcL
Hb	15.5 g/dL	14.0 - 18.0 g/dL
MCV	83.3 fL	80.0 - 99.0 fL
Platelet	150 K/uL	150 - 450 x 10 (3)/mcL
Neutrophil %	78.7	44.0 - 70.0%
Lymphocyte %	11.4	20.0 - 45.0%
Monocyte %	8.4	2.0 - 10.0%
Eosinophil %	0.3	1.0 - 4.0%
Basophil %	0.4	0.0 - 2.0%
Immature granulocytes, absolute %	0.8	0.0 - 2.0%
Neutrophil absolute	5.77	2.10 - 7.60 x 10 (3)/mcL
Lymphocyte absolute	0.84	1.00 - 4.90 x 10 (3)/mcL
Monocyte absolute	0.62	0.10 - 1.10 x 10 (3)/mcL
Eosinophil absolute	0.02	0.10 - 0.40 x 10 (3)/mcL
Basophil absolute	0.03	0.00 - 0.20 x 10 (3)/mcL
Immature granulocytes, absolute	0.06	0.00 - 0.20 x 10 (3)/mcL
ALT	52	0 - 41 U/L
AST	34	0 - 50 U/L
Total bilirubin	0.30	0.00 - 1.20 mg/dL
Direct bilirubin	0.10	0.00 - 0.20 mg/dL
Indirect bilirubin	0.2	0.0 - 1.0 mg/dL
ALP	78	40 - 129 U/L
Ova and parasite screen stool	Moderate *Endolimax nana* cysts, few *Blastocystis hominis* cysts	
Quantiferon TB	Negative	
HIV	Negative	
Cysticercosis antibodies	Negative	
*Strongyloides* antibodies	Negative	

**Figure 1:**
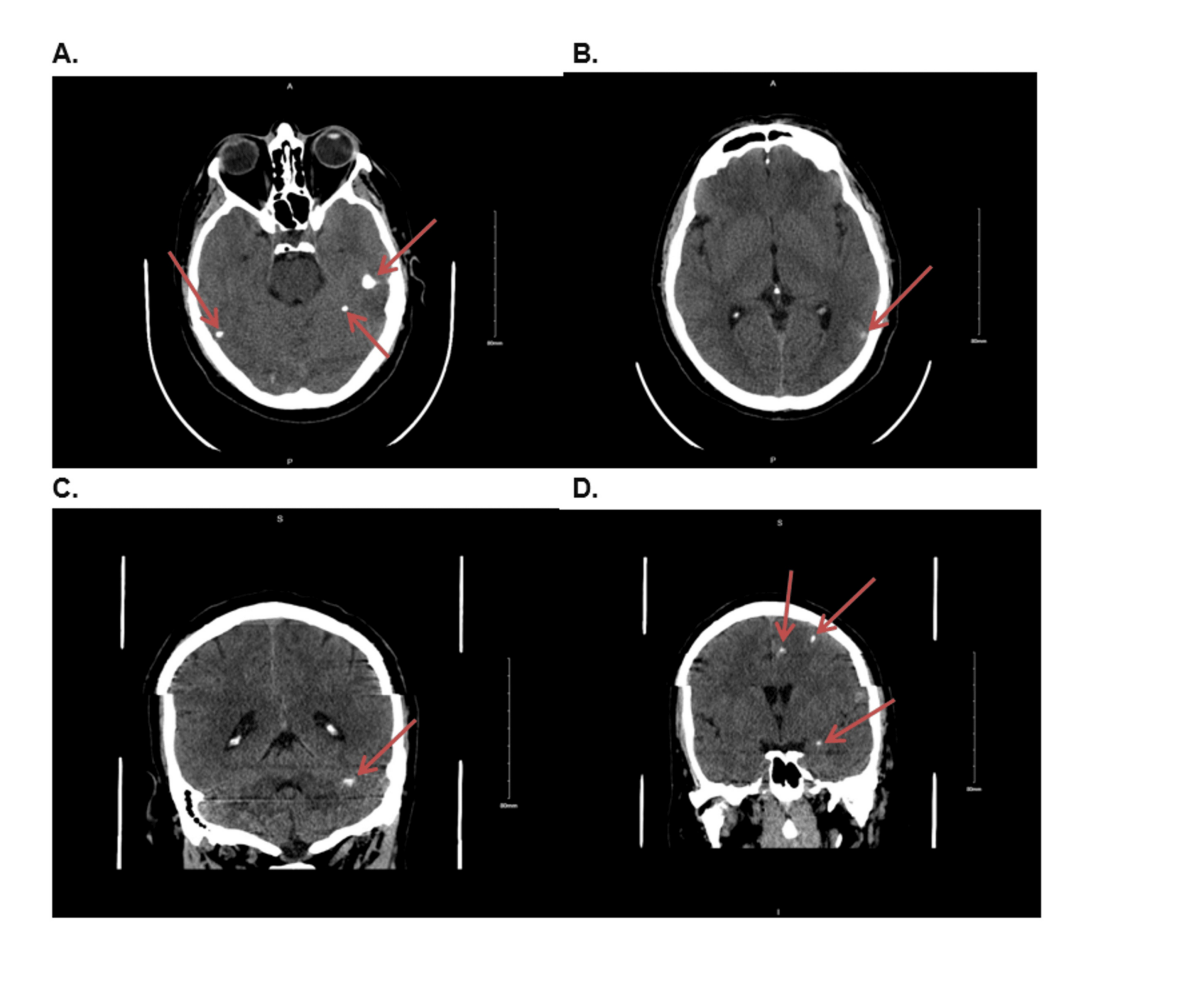
A-D: CT head without contrast showing multiple partially calcified lesions throughout the brain. The lesions appear hyperdense, representing calcified, nonviable cysts, which correspond to the late stage of neurocysticercosis.

**Figure 2:**
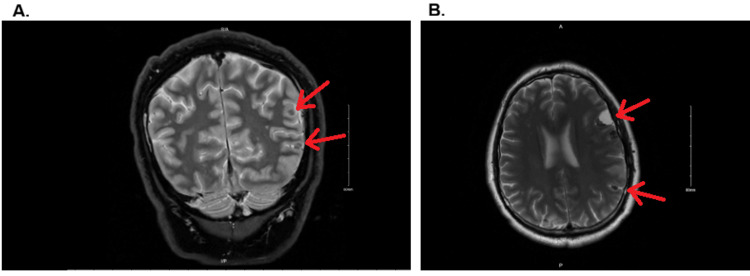
A-B: MRI brain with and without contrast showing peripherally enhancing cystic lesions in the left frontal cortex and right posterior parietal cortex, consistent with the vesicular (early) stage of neurocysticercosis. The lesions exhibit mild peripheral enhancement, small nodular components, and minimal surrounding edema. Smaller calcified granulomas, indicative of the calcified (late) stage, are also seen.

CT head without contrast demonstrated multiple intracranial calcified lesions concerning for NCC (Figure 1). MRI brain with and without contrast confirmed multiple cystic and calcified lesions (Figure 2).

Despite negative serology, a diagnosis of NCC was made based on the patient’s clinical presentation, his history of immigration from an endemic region, and neuroimaging findings consistent with cystic lesions, as outlined in the Del Brutto diagnostic criteria.

The patient was started on dual antiparasitic therapy with albendazole and praziquantel, chosen for its effectiveness in treating severe or complicated parasitic infections due to its complementary action in targeting different stages of the parasite’s lifecycle. This was complemented by a short, 10-day course of corticosteroids to manage inflammation and levetiracetam for seizure prophylaxis. This combination improves outcomes, reduces complications, and helps prevent seizure recurrence. Upon follow-up with the infectious disease (ID) clinic, the patient reported good adherence to the prescribed medications. However, due to abnormal liver function tests, albendazole and praziquantel were temporarily discontinued. These medications were later resumed after liver function improved. During a subsequent neurology follow-up, the patient reported no recurrence of seizures. Levetiracetam was continued, and a follow-up brain MRI and electroencephalogram (EEG) were ordered for further evaluation.

## Discussion

NCC, caused by *T. solium* larval cysts, is a leading cause of acquired epilepsy worldwide [10]. Although the same parasite causes taeniasis, NCC arises predominantly through fecal-oral transmission of *T. solium* eggs rather than ingestion of cysticerci [10]. After ingestion, eggs hatch in the intestine to oncospheres, which penetrate the mucosa, disseminate via the bloodstream, and develop into cysticerci in various tissues, including the CNS, skeletal muscle, subcutaneous tissue, and the eye [10,11].

These cysts may remain clinically silent for years, with minimal inflammatory response until degeneration occurs [11]. Within the CNS, cysticerci may localize to brain parenchyma, subarachnoid space, or ventricles, sometimes affecting multiple regions simultaneously. Clinical manifestations depend on both cyst location and developmental stage. In the viable (vesicular) stage, larvae often provoke little inflammation, but when cysts degenerate (colloidal stage), they trigger an inflammatory response that can lead to seizures [10].

Over time, cysts may shrink, involute, or calcify. However, calcified lesions are not always inert: a substantial subset may remain epileptogenic, likely due to intermittent perilesional inflammation, transient edema, or gliosis, processes increasingly documented in calcified NCC patients [12-14].

Diagnosis relies on neuroimaging in combination with clinical evaluation and, when available, immunologic or serologic testing [10]. The Del Brutto diagnostic criteria for NCC classify findings into absolute, major, and confirmative neuroimaging, minor neuroimaging, and clinical/exposure categories, enabling clinicians to establish a definitive or probable diagnosis based on combinations of these features [11]. Absolute criteria include histologic demonstration of the parasite from a CNS biopsy, visualization of subretinal cysticerci, or identification of the scolex on imaging. Major neuroimaging criteria include cystic lesions without a visible scolex, enhancing lesions, and characteristic parenchymal calcifications, while confirmative criteria comprise resolution of cysts after cysticidal therapy, spontaneous resolution of small enhancing lesions, or migration of ventricular cysts on sequential imaging. Clinical/exposure criteria encompass serologic detection of anticysticercal antibodies or antigens, evidence of systemic cysticercosis or a household *Taenia* carrier, suggestive clinical manifestations, or residence in/immigration from endemic areas. A definitive diagnosis can be reached with one absolute criterion, or by combining two major neuroimaging criteria plus clinical/exposure evidence, one major and one confirmative neuroimaging criterion plus clinical/exposure evidence, or one major neuroimaging criterion plus two clinical/exposure criteria. Cases with more limited findings may qualify as a probable diagnosis under less stringent combinations [11].

Prognosis is influenced by cyst burden and location; patients with a small number of parenchymal lesions generally have a more favorable outcome, whereas extraparenchymal involvement (e.g., subarachnoid or intraventricular) carries higher morbidity [10,11].

Therapy for NCC typically combines cysticidal agents, such as albendazole or praziquantel, corticosteroids to reduce inflammation, and antiepileptic medications for seizure control [10,11]. Corticosteroids are often initiated prior to antiparasitic therapy to prevent exacerbation of inflammatory responses, though timing can vary in clinical practice [10,11]. In our patient, this approach was applied alongside seizure prophylaxis.

Interestingly, stool studies revealed *B. hominis* and *E. nana* cysts. While these protozoa are not causally related to *T. solium*, their presence reflects shared fecal-oral transmission and may indicate environmental exposure or poor hygiene. The absence of gastrointestinal symptoms illustrates that co-infections may be clinically silent yet epidemiologically informative.

This case highlights several important points: (1) Latency of NCC - exposure to risk factors, including living in an endemic area and consuming pork, may have occurred many years before symptom onset, demonstrating that NCC can remain dormant for long periods [10,11]; (2) Social determinants of health - factors such as geographic origin, immigration status, and access to healthcare can influence disease recognition, management, and outcomes [12]; (3) Early detection and prevention - awareness of these factors is critical for timely diagnosis, patient education, and prevention of NCC among patients from endemic regions and underserved populations [10,12]. Recognition of NCC in patients from endemic areas, even decades after exposure, facilitates timely treatment and improved outcomes [15].

## Conclusions

NCC, caused by *T. solium*, is a leading cause of acquired seizures in endemic regions. While rare in the United States, increasing immigration from endemic areas has led to a rise in cases, highlighting the importance of considering NCC in patients with new-onset seizures, especially immigrants. In our patient, who had no recent travel or pork consumption history, the combination of his presentation with generalized tonic-clonic seizures and neuroimaging suggestive of cystic lesions was crucial in diagnosing NCC and initiating appropriate antiparasitic therapy and seizure prophylaxis. A thorough social history is essential for identifying potential exposures, and clinicians should maintain a broad differential when diagnosing new-onset seizures to ensure timely intervention, which can significantly improve outcomes and prevent complications.
